# Matrix Models for Size-Structured Populations: Unrealistic Fast Growth or Simply Diffusion?

**DOI:** 10.1371/journal.pone.0098254

**Published:** 2014-06-06

**Authors:** Nicolas Picard, Jingjing Liang

**Affiliations:** 1 UPR Biens et services des écosystèmes forestiers tropicaux (BSEF), Centre de coopération internationale en recherche agronomique pour le développement (CIRAD), Montpellier, France; 2 Division of Forestry and Natural Resources, West Virginia University, Morgantown, West Virginia, United States of America; University of California-Irvine, United States of America

## Abstract

Matrix population models are widely used to study population dynamics but have been criticized because their outputs are sensitive to the dimension of the matrix (or, equivalently, to the class width). This sensitivity is concerning for the population growth rate (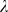) because this is an intrinsic characteristic of the population that should not depend on the model specification. It has been suggested that the sensitivity of 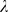 to matrix dimension was linked to the existence of fast pathways (i.e. the fraction of individuals that systematically move up a class), whose proportion increases when class width increases. We showed that for matrix population models with growth transition only from class 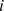 to class 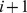, 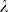 was independent of the class width when the mortality and the recruitment rates were constant, irrespective of the growth rate. We also showed that if there were indeed fast pathways, there were also in about the same proportion slow pathways (i.e. the fraction of individuals that systematically remained in the same class), and that they jointly act as a diffusion process (where diffusion here is the movement in size of an individual whose size increments are random according to a normal distribution with mean zero). For 53 tree species from a tropical rain forest in the Central African Republic, the diffusion resulting from common matrix dimensions was much stronger than would be realistic. Yet, the sensitivity of 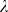 to matrix dimension for a class width in the range 1–10 cm was small, much smaller than the sampling uncertainty on the value of 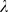. Moreover, 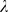 could either increase or decrease when class width increased depending on the species. Overall, even if the class width should be kept small enough to limit diffusion, it had little impact on the estimate of 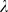 for tree species.

## Introduction

To model the dynamics of a population, there are two main options depending on the level of the population: individual-based models, where the trajectory of every individual is monitored; and distribution-based population models, where individual attributes are summarized by their population-level distribution [1], [2]. Among the latter, four types of models can be distinguished depending on whether the distribution and time are modeled as continuous or discrete: matrix population models (discrete distribution and discrete time [3]), integral projection model (continuous distribution and discrete time [4]), continuous-time Markov chain (discrete distribution and continuous time, e.g. [5]), and partial differential equations (continuous distribution and continuous time [6]). There are two diverging opinions on the choice of a modeling approach. On one hand, some have highlighted the advantages and limitations of each approach, thus suggesting that some approach may intrinsically be superior to the others. For instance, matrix population models have been criticized for the arbitrariness of the class division [7]–[10] and integral projection model put forward as a solution to this issue [11]–[13]. On the other hand, others have put the emphasis on the theoretical connections that exist between all these modeling approaches [14]–[18], thus suggesting that the choice of a modeling approach should be a pragmatic choice that marginally affects the predictions [3,§8.5].

Matrix models have been criticized for their inability “to incorporate variation among individuals within a size category” [7, p.346]. Because matrix models operate on size- (or age-, or stage-) structured populations, differences of growth among individuals due to size are accounted by the model, but size may indeed be an incomplete predictor of growth. Nevertheless, in this case, additional predictors of growth can be added as structuring variables of the population, with a subdivision of the categories of the transition matrix [3, §8.4] [19]. To address autocorrelation in growth, second- (or higher-) order Markov chains (and corresponding transition matrices) can also be considered [20]. Finally, residual error in growth which results from random variability in individual growth can also be addressed in matrix modeling using random shocks [21] or as a diffusion process (i.e. by adding transition rates off the main diagonal of the transition matrix [3, p.199]).

Second, matrix models have been criticized because their outputs (population growth rate 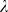, i.e. the temporal rate of change of the population number of individuals on the long term; elasticities, i.e. the relative rate of change of an output with respect to a parameter; age estimates) are sensitive to the dimension of the matrix (or, equivalently, to the width of the size classes for a given range of size) [8], [9], [13], [22]. This sensitivity to matrix dimensionality is concerning when the outputs are intrinsic population characteristics that should be defined irrespective of the mathematical model of population dynamics and, thus, irrespective of the particular division of size (or age) into classes. The dependence of elasticities on matrix dimensionality is consistent with the fact that the relative importance of growth compared with stasis (i.e. remaining in a size-class for more than one time step) changes with the class width [23]. Moreover, the dependence of the population growth rate 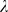 on matrix dimensionality has been questioned by other studies [24], [25]. In some cases, the influence of matrix dimensionality on model outputs has been investigated using different populations with different models fitted to them [10], [13], [26]. In this case, the effect of matrix dimension on model outputs can be confounded with the effect of population differences.

As a possible explanation of the dependence of 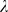 on matrix dimensionality, Zuidema et al. wrote [7, p.346]: “As the transition probabilities in a matrix model depend only on the current situation, there is no obstruction for unrealistically fast pathways through the life cycle. For instance, in matrix models with 10-cm-wide diameter categories and small progression probabilities, a small fraction may reach 50 cm diameter in five time steps, something that is clearly impossible biologically (and physically). This fraction contributes strongly to population growth and probably causes the high estimates of 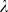 for small matrix models.” Because the population growth rate 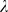 is an intrinsic characteristic of the population that is often used in population viability analysis and that should not depend on the model specifications, understanding why 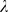 depends on matrix dimensionality in matrix population models would indicate to which extent different modeling approaches are reconcilable. In case of significant dependence, it should provide guidance to the modelers about which modeling approach to use.

In this study, we will assess the dependence of the population growth rate 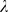 on class width, and how fast pathways (i.e. the fraction of individuals that systematically move up a class at each time step) possibly contribute to this dependence. We will show that if there are indeed fast pathways, there are also in about the same proportion slow pathways, i.e. the fraction of individuals that remain in the same class for a long time. As a consequence, fast pathways do not directly bias growth rates towards higher than expected values, but rather, in combination with slow pathways, act as a diffusion process with limited impact on the estimate of 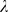. Diffusion here is defined as the movement in size of an individual whose size increments are random following a normal distribution with mean zero. At the population level, these individual random walks flatten the size distribution and make it uniform (in the statistical distribution sense). This study is based on a data set from a tropical rain forest in central Africa, including different tree species whose dynamics are modeled by a Usher transition matrix (i.e. a transition matrix with non null elements on its main diagonal, on its lower subdiagonal and on its first row only).

## Materials and Methods

### The M’Baïki Forest

The M’Baïki experimental site is located in the south of the Central African Republic (3

54′N, 17

56′E), at the northern limit of the rain forest of the Congo basin. It is dedicated to studying the effects of logging damage on stock recovery [27] and lies in a *terra firme* rain forest. The experimental design of the site consists of two blocks of three and one block of four 300
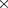
300-m permanent sample plots with a 50-m inner buffer zone. In each central 200
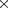
200-m square, all trees over 10-cm diameter at breast height (dbh) were identified and georeferenced. Since 1982, girth at breast height, standing deaths, treefalls, and newly recruited trees over 10-cm dbh have been monitored annually except in 1997, 1999 and 2001. Between 1984 and 1985, two silvicultural treatments were applied: three plots (including the buffer zone) were logged, and four plots were logged and thinned. The three remaining plots were left as controls. For this study, we used the data of the control plots only, between 1982 and 2006 with a time step of 2 years. In total, 66,749 tree records in 53 species were used in this study.

### Changing the Dimension of a Transition Matrix

Given a transition matrix 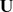 for a size-structured population with 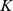 size classes, different techniques have been proposed to derive a transition matrix 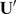 for 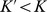 size classes. When the data used to fit 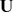 are available, a data-driven approach consists in refitting the transition matrix using these data and 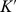 classes. This approach has the advantage that the possible resulting change in the estimate of the population growth rate 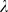 readily corresponds to what is observed when fitting the matrix model. The limitation is that it is not possible to disentangle what is specifically due to the data set used and what is due to the properties of matrix modeling in general.

A second technique consists in computing the transition rates of the 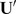 matrix from those of 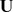. For instance, when 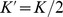 and when combining every two successive classes 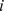 and 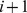 into a single one 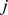, it has been proposed to compute the upgrowth transition rate 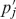, the mortality rate 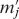 and the recruitment rate 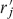 of 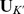 as [8], [9]:
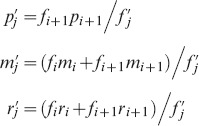
(1)where 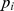, 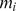 and 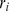 are the transition rates of 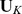 for growth, mortality and recruitment respectively, 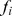 is the number of individuals in size class 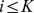, and 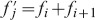 is the number of individuals in size class 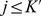. This approach has the advantage that no additional information beyond the population-level characteristics are needed to change the size of the transition matrix. A first limitation is that this technique can be used only when 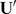 is nested into 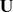 (i.e. when the 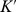 classes are obtained by merging together some of the 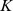 classes). A second limitation, more theoretical, is that the relationships between the transition rates of 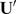 and those of 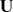 depend on the number 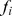 of trees in the classes. Because 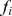 changes with time, this implies that the 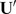 matrix derived from 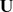 will not be the same depending on the time step considered (even if 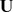 is stationary), which is not consistent. Moreover, other algebraic relationships than (1) could be used to collapse 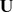 into 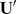. In particular, it is mathematically feasible to collapse a transition matrix into a smaller matrix while maintaining the same dominant eigenvalue and eigenvectors [10], [25], [28]–[30]. If such algebraic relationships were to be used rather than (1) to collapse matrices (and there is no theoretical reason for not doing it) then, by construction, 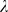 would not depend on the class width.

A third technique to change the dimension of the transition matrix dates back to [14], [15] and is based on the connection between matrix population model and continuous partial differential equations [3,§8.1.4]. In particular, it has been the basis for optimizing the width of size classes in matrix models for size-structured populations [31], [32], considering that the matrix model is a discrete approximation of a continuous partial differential equation with a bias/variance trade-off to optimize. It has the advantage that it can deal with any change of the class limits in a theoretically consistent manner [33]. It has the limitation that size must be continuous and that a model of continuous-size dynamics must be assumed as a prerequisite. The matrix population model can be seen as a discretization of a McKendrick partial differential equation [16], [34]–[36]:
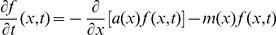
(2)


with the boundary condition:
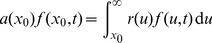
(3)where 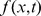 is the continuous size distribution at time 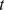, such that the number of individuals with a size between 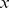 and 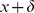 for any infinitesimally small 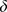 is 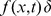, 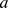 is the size growth rate, 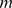 is the mortality rate, 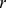 is the recruitment rate, and 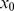 is the minimum size for inventory. Let us consider a numerical scheme to solve (2) [37, chapter 20]. Size is discretized using a size spacing of 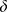: 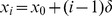 where 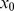 is the minimum size and 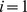,…, 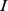. Time is discretized using a time spacing of 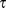: 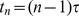 where 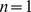,…, 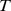. Let 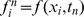 be the discretized value of the size distribution. A forward-time left-size differencing scheme for (2)–(3) is:



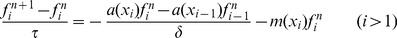
(4)

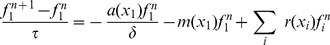


which can be written as:
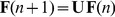
(5)where 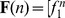,…, 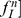 is the vector of length 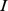 that contains the number of individuals at discrete time 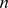 in each size class with width 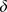 and lower bound 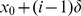, and 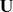 is a Usher transition matrix:
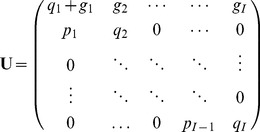
(6)where the stasis rate 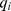 (i.e. the probability for an individual to stay alive in class 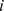 between two consecutive time steps), the upgrowth transition rate 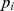 (i.e. the probability for an individual to grow up from class 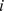 to class 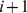), and the recruitment rate 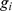 are given by:



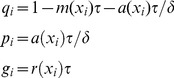
(7)
Equation (5) corresponds to the Usher matrix model for size-structured populations [38], [39], which has developed in forestry independently from the McKendrick equation (e.g. [40]–[42]). Given an individual-based model of size growth 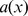, an individual-based model of death probability 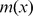, and an individual-based model of recruitment 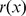, equation (7) defines the transition rates of the Usher matrix 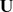 for any partition of size into classes of width 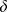. Notice that 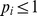 for all 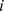 implies:
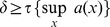
(8)


which corresponds to the Usher [38] hypothesis that individuals cannot grow by more than a single class in one time step.

### Population Growth Rate

The asymptotic population growth rate, 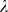, is the dominant eigenvalue of the transition matrix 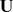 as given by (6) [3]. In the general case, there is no explicit expression for 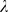. However, in the particular case of a Usher transition matrix with constant mortality rate 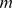 and constant recruitment rate 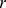 (i.e. 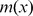 and 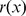 do not depend on size 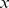), and irrespective of the variations of the growth rate 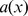 with size, there is an analytical expression for 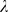 [24], [43], [44]:
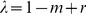
(9)which mathematically proves that 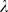 does not depend on the class width in this case. This result is also valid when transition matrices are collapsed using (1), because 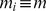 and 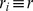 for all 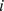 imply 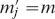 and 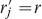 for all 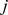. A corollary of this result is that variations of 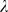 with class width can occur only if the mortality rate or the recruitment rate varies with size. Therefore, in this study, we will only consider matrix models with either size-dependent mortality or size-dependent recruitment.

When dealing with forest ecosystems, due to the complexity of sexual and asexual reproductions and variability of elapsed time for germinated seeds to become recruited trees, it is not possible to assign a newly recruited tree as originating from a given size class [13]. Therefore, when dealing with forest dynamics, an average recruitment rate (the same for all size classes) is generally estimated as the ratio of the number of newly recruited trees over the number of trees at the previous time step (see [22], [45] for exceptions). If such is the case, variations of 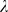 with class width can occur only if the mortality rate varies with size.

Practically, to assess how 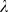 varied with class width for the tree species at M’Baïki, the following analyses were performed. Trees were classified into diameter at breast height (dbh) classes with equal width 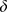, ranging from a minimum dbh for inventory of 10 cm to a maximum dbh of 150 cm. The number 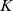 of dbh classes correspondingly varied proportionally to 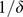. The time step of the matrix model was 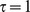 year. For each species with at least 300 observations, a constant dbh growth rate 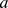 was estimated from the M’Baïki data base as the empirical mean of the dbh increments (including negative increments) over 2 years divided by this period of 2 years. The variations of the growth rate with dbh were not considered here because they are not a condition for 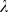 to vary with class width 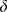. A constant recruitment rate was estimated for each species as the ratio of the number of newly recruited at year 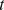 over the number of living trees at year 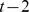, divided by this period of 2 years.

The dependence of mortality on dbh was necessarily accounted because it is a condition for 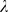 to vary with class width 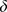. The tree mortality rate was modeled for each species as a function of tree dbh using one of the three following models [46], [47]:
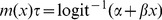
(10)

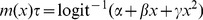
(11)

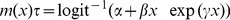
(12)where 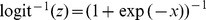 is the inverse logit function and 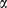, 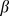, 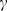 are parameters to estimate. Models (10) and (11) were fitted using the generalized linear model (command glm in R software) whereas model (12) was fitted using the generalized non-linear model (package gnm in R software). The three models were compared using the Akaike Information Criterion (AIC) and the one with the lowest AIC was retained. Given the growth rate 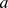, the mortality rate 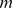 and the recruitment rate 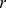 for each species, the class width 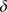 was changed from 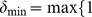, 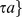 cm to 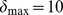 cm; for each value of 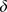, the Usher transition matrix was computed using (7), and the population growth rate 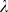 was computed as the dominant eigenvalue of this matrix.

The variations of 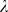 with 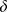 were compared to the sampling variability of 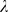 for the smallest class width 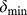 (i.e. for the matrix model that is the closest to the McKendrick equation (2)). The sampling variability of 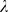 represents the uncertainty on 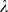 due to the finiteness of the data set used to estimate 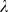. For each species, a 95% confidence interval of the estimate of 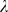 for the smallest class width 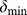 was computed using 500 bootstrap replicates [48], [49].

### Fast and Slow Pathways

The McKendrick equation corresponds to a propagation of the diameter distribution at a speed defined by 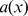. Hence, implicitly with the McKendrick equation, all trees with the same diameter grow at the same rate and there is no fast pathway. If the Usher matrix model was an exact scheme to solve the McKendrick equation, there would be no fast pathway either. Therefore, the fast pathways depicted by Zuidema et al. [7] correspond to the approximation brought by the discretization of (2) into (5).

When focusing on the upgrowth part of the McKendrick equation (i.e. setting the mortality and the recruitment rates to zero), a von Neumann stability analysis [37,§20.1.1] shows that the Usher matrix model is a stable numerical scheme to solve the McKendrick equation provided that condition (8) is met. Therefore, the Usher condition that no individual can grow by more than one class in a single time step identifies with the Courant-Friedrichs-Lewy condition in numerical analysis. The von Neumann stability analysis also shows that the Usher matrix model is numerically dissipative unless 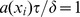 for all 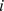. This means that in the Fourier transform of the distribution 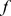, all terms with a wave number 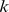 such that 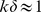 will be inaccurately calculated. This dissipative effect of the Usher scheme can be intuitively understood by considering that the numerical scheme (4) can be rewritten as:
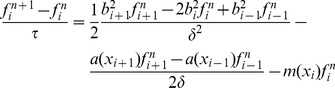
(13)where 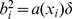 for all 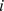. The numeric scheme (13) corresponds to the forward-time centered-size differencing scheme for the Fokker-Planck equation:
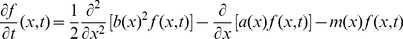
(14)with 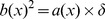. When compared to the McKendrick equation (2), the Fokker-Planck equation has an additional term (the second-order partial derivative in (14)) that corresponds to diffusion.

As a first result, the fast pathways pointed out by [7] can be interpreted as a diffusion process and go along with slow pathways, i.e. individuals that remain in the same size class longer than expected. The diffusion process has a simple biological interpretation and may be a desirable feature [40], [42]. It relates to the individual variability in growth. More precisely, 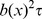 can be interpreted as the variance of the individual size increments during an infinitesimally small time interval 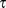 for trees with size 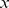. Hence, the diffusion process will be realistic provided that 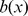 is a realistic model for the standard deviation of annual tree size increments. The Usher scheme implies that 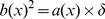. Hence, the diffusion generated by the Usher scheme will remain biologically realistic as long as
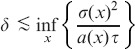
(15)where 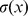 is the standard deviation of tree size increments during the time interval of trees 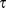 years.

To visualize fast and slow pathways, we considered the transient dynamics of a single even-aged cohort of trees uniformly distributed between 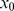 and 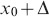 where 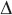 is the dbh amplitude of the cohort, i.e.: 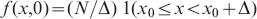, where 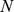 is the initial number of trees and 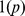 is the indicator function of proposition 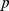 (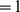 is 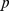 is true and 0 if 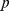 is false). To focus on the transient dynamics of this cohort, recruitment was set to zero (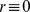). In the particular case where the growth rate 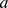 is constant, the analytical solution of the McKendrick equation (2) is known [16, p.45] [36] and corresponds at time 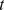 to a displacement of the cohort by a dbh 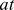 with an attenuation of the number of trees with dbh 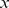 by 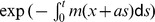, i.e.:
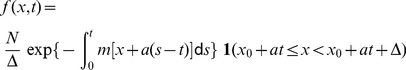
(16)


In the particular case where 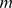 is given by model (10),
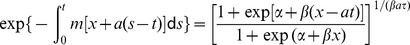



By comparing the exact solution (16) of the McKendrick equation to the prediction of the Usher matrix model, fast and slow pathways due to discretization can be identified. We calculated the proportion of fast pathways as the proportion of 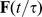 that was above 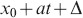, and the proportion of slow pathways as the proportion of 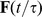 that was below 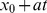.

## Results

### Variations for *Celtis zenkeri*

To illustrate the dependence of the population growth rate 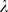 on the class width, we first describe the variations of 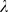 for just one species taken from the Mbaki data set. We chose *Celtis zenkeri* Engl. (Ulmaceae) because it was the most abundant species of the data set (with 7295 observations between 1982 and 2006) and representative of the most commonly observed pattern of variation of 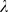. Its average growth rate was 0.263 cm yr^−1^ (standard deviation: 0.30 cm yr^−1^). Its recruitment rate was 1.028% yr^−1^. Its mortality rate was best modeled by model (10) with 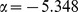 (std. dev.: 0.272) and 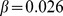 (std. dev.: 0.009). Therefore, the mortality rate for *C. zenkeri* was an increasing function of tree dbh, ranging from 0.6% yr^−1^ for a dbh of 10 cm to 2.3% yr^−1^ for a dbh of 62 cm (that is the 99.5% percentile of dbh for *C. zenkeri* at M’Bïaki).

**Figure 1:**
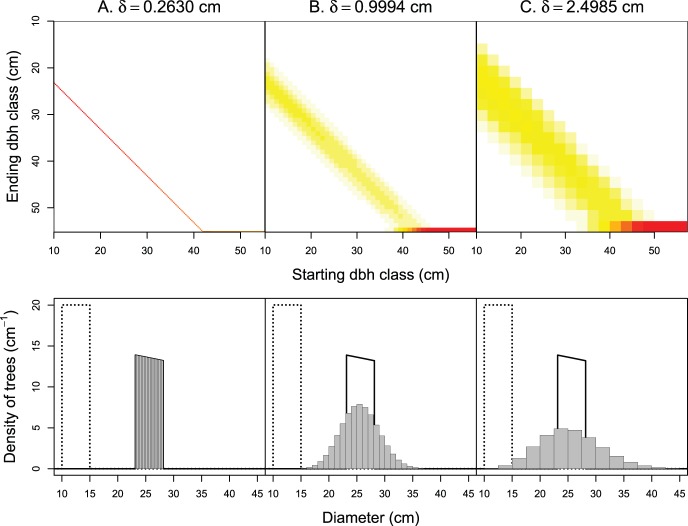
Growth of a cohort of 100 *Celtis zenkeri* trees with a uniform initial diameter distribution. Projection time is 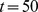 yr. The time step of the matrix model is 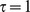 yr and the class width is column-wise (A) 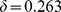 cm, (B) 0.9994 cm, and (C) 2.4985 cm. The top panel shows images of the transitions matrices between the initial and final times (i.e. the annual transition matrices raised to the power of 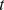), where the starting class is column-wise, the ending class is row-wise, and the transition rates between classes are shown using heat colors (from white = zero to red = the highest values). The bottom panel shows the predicted dbh distributions: dotted line = initial dbh distribution (uniform across 10–14.997 cm); solid line = final dbh distribution according to the McKendrick continuous model; shaded bins = final dbh distribution according to the Usher matrix model.

To illustrate fast and slow pathways, we considered the dynamics of an even-aged cohort of 100 trees uniformly distributed between 10 and 15.0 cm at 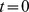. When 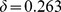 cm and 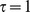 yr, the condition 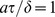 was met and the Usher scheme for growth was not dissipative (Figure 0A): there were neither fast pathways nor slow ones in this case. With the exception of the last class, non-null transition rates defined one-to-one connections between classes. The only difference between the exact solution of the McKendrick equation and the Usher model followed from the difference between 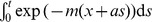 (for the exact solution) and 
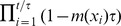
 (for the Usher model) for the attenuation of the number of trees. For *C. zenkeri*, this difference was actually so small that it is not visually perceptible in Figure 1A.

When 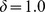 cm and 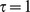 yr, 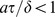 and the Usher scheme became dissipative (Figure 1B): there were some fast and slow pathways in this case. Several classes at initial time contributed to the number of trees in any class at final time. For *C. zenkeri*, the proportion of slow pathways (22.8%) was greater than the proportion of fast pathways (15.9%). If the mortality rate 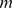 was constant, then the proportion of slow pathways would have been exactly equal to that of fast pathways. The class width 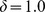 cm brought the same diffusion as a random growth with standard deviation 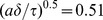 cm yr^−1^, which is greater than the observed standard deviation of the growth rate (0.30 cm yr^−1^). The dissipative effect of the Usher scheme increased as the class width increased from 1.0 to 2.5 cm (Figure 1C). For this latter class width, the proportions of slow and fast pathways were 30.9% and 20.1%, respectively, and the dissipation was equivalent with that produced by a random growth with standard deviation 0.81 cm yr^−1^.

**Figure 2:**
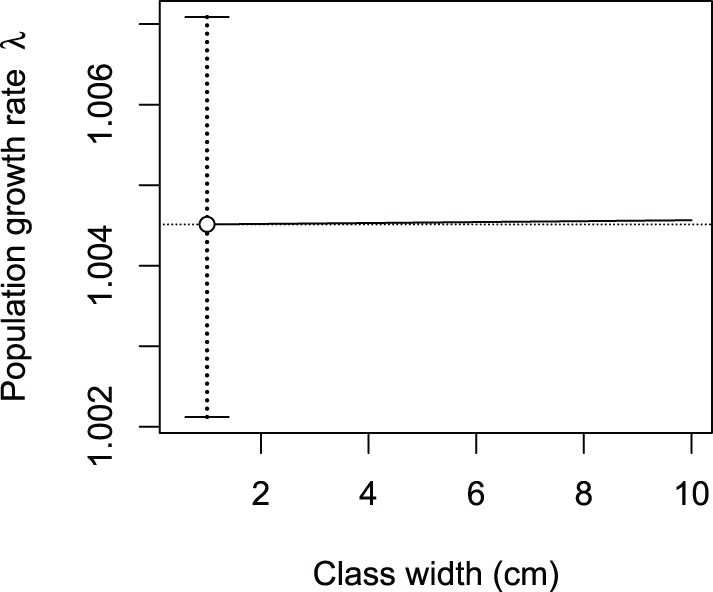
Variations of the population growth rate 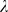 of *Celtis zenkeri* with class width 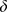. 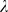
 is computed using a Usher matrix model. It increases with 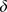 (solid line), but this increase is negligible on the range from 1 to 10 cm as compared to the 95% confidence interval of the estimate of 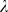 (shown by the dot and whiskers). The horizontal dotted line corresponds to the value of 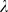 for 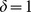 cm.

The population growth rate of *C. zenkeri* increased from 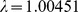 for a class width of 1 cm to 1.00457 for a class width of 10 cm. In comparison, the 95% confidence interval of the estimate of 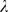 for 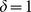 cm was 1.00212–1.00709. Therefore, the amplitude of the 95% confidence interval of the estimate of 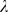 for 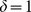 cm was 96 times greater than that of the variations of 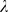 for 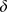 varying from 1 to 10 cm (Figure 2), even though *C. zenkeri* was the species with the largest number of observations and the narrowest 95% confidence interval.

### Variations Across Species

After excluding those species for which the mortality rate did not significantly vary with dbh (and thus with a population growth rate 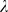 that did vary with 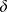), there were 53 species left. Models (10), (11) and (12) for mortality were selected for 85%, 9% and 6% of the species, respectively (Table S1). For 74% of the species, the population growth rate 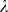 increased with class width 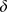; for 24% of the species, 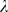 decreased with 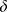; and for 2% of the species, the change of 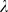 when 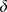 varied from 1 to 10 cm was less than 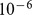. There was no one-to-one relationship between the direction of change of 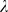 and the shape of the mortality model, with all combinations of mortality model and direction of change of 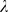 being observed. Nevertheless, when the mortality rate 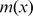 was an increasing function of dbh 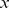, 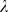 most often (but not always) increased with 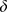.

**Figure 3:**
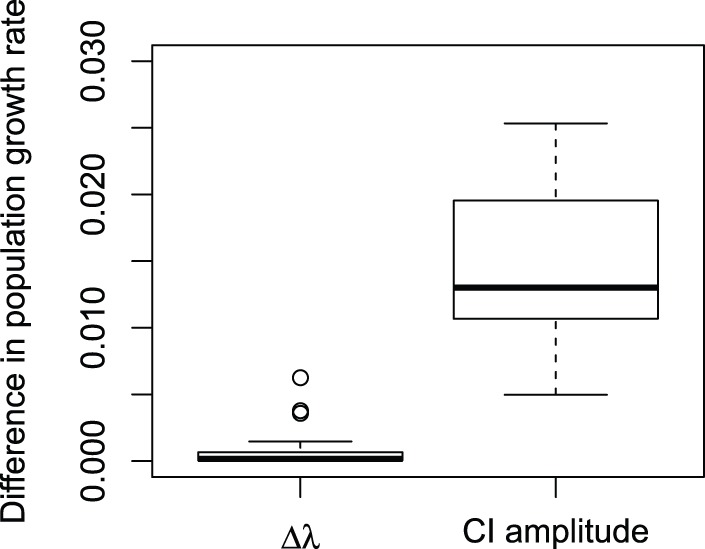
Distribution across 53 species of the amplitude of variations of the population growth rate 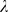. Left boxplot: variations of 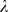 when the class width 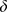 varies from 1 to 10 cm, where 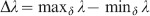. Right boxplot: amplitude of the 95% confidence interval of the estimate of 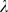 for 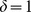 cm.

On average across species, the amplitude of the 95% confidence interval of the estimate of 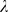 for 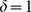 cm was 31 times greater than that of the variations of 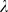 for 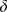 varying from 1 to 10 cm (Figure 3). No species had a population growth rate 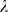 for 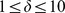 cm that went outside the 95% confidence interval of the estimate of 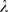 for 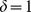 cm.

**Figure 4:**
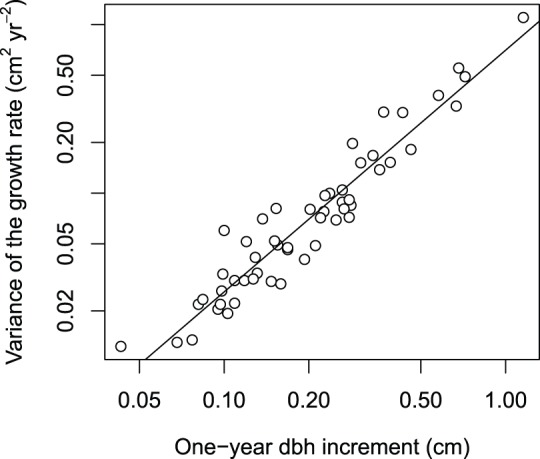
Variance of the dbh growth rate versus one-year dbh increment for 53 tree species. Notice that axes are in logarithmic scale. The line is the regression line on log-transformed data.

The relationship across species between the variance 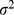 of the growth rate and the one-year dbh increment 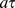 could be modeled by a power relationship: 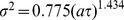 (Figure 4). Combining the Usher/Courant-Friedrichs-Lewy condition (8), condition (15) and this power relationship gives the following approximate interval for the class width:
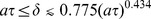



Only very fast growing species, with a dbh growth rate greater than 0.64 cm yr^−1^, cannot meet this condition. At M’Baïki, only four species (*Musanga cecropioides* R. Br., *Trilepisium madagascariense* DC., *Macaranga paxii*, and *Ricinodendron heudelotii*) had a mean growth rate greater than 0.64 cm yr^−1^. For all other species (92% of the species), it would be possible to set a class width that is consistent with the variability in growth of the species. However, the resulting class width (less than 0.64 cm for a time step of 1 year) would be much less than the class widths commonly used in matrix modeling for forest dynamics.

## Discussion

### Is 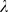 Sensitive to Class Width?

The Usher matrix model can be seen as a discrete approximation of a continuous-size distribution model. This discretization induces a diffusion, with fast pathways (i.e. fractions of trees that grow up classes faster than expected), but also slow pathways (i.e. fractions of trees that remain in the same class longer than expected). Diffusion in itself is appropriate since it corresponds to the individual variability in growth. Hence, slow pathways represent the fraction of individuals with the lowest growth (possibly including those with negative growth). There are also instances when fast pathways are appropriate, in particular for stage-structured populations when individuals are able to skip intermediate stages in their ontogenic development [50]. However, in size-structured populations, the strength of diffusion is directly related to the class width, and the class widths often used in matrix modeling in forestry (often in the range 3–10 cm for dbh; [51]) induces a diffusion that is much stronger than that solely due to the individual variability in growth.

Although the diffusion due to the discretization in classes was much greater than what would be realistic, the matrix model predictions of 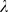 at M’Baïki were particularly robust to changes of the class width 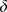, for variations of 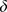 as large as from 1 to 10 cm. The median of the difference 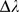 between the maximum value of the population growth rate and its minimum for a class width ranging from 1 to 10 cm was 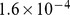 at M’Baïki. In comparison, Enright et al. [8] found a difference 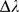 of zero for three tree and a grass species (including an imaginary tree species), and a difference of 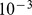 for the tropical conifer *Araucaria cunninghamii*. Ramula and Lehtilä [9] found differences 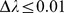 for 19 tree species and significantly larger differences for 18 herbaceous species. Zuidema [22] found a maximum difference of 0.009 for four tree species and 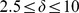 cm.

Higher variations of 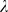 with class width may be obtained when the recruitment rates are not constant with size [8], [9]. Ramula and Lehtilä [9] reported that changes in 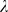 were significantly larger for herbaceous than for woody species, maybe because the former have much less classes that are based on development stage rather than on size. Transition matrices do not have the same structure for herbs and trees, with higher recruitment for herbs and more frequent retrogression [52]. Therefore, the robustness of 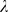 to class width that was observed at M’Baïki for tree species is also linked to the specific structure of the transition matrix and may not be extrapolated to other types of transition matrices.

The direction of variation of 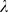 with the class width depended in a complex and non-systematic way on the demographic rates (growth rate, mortality rate, recruitment rate). The population growth rate at M’Baïki could increase or decrease as the class width increased, whether or not the mortality was an increasing function of size. Ramula and Lehtilä [9] also observed that 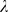 could increase or decrease when matrix dimensionality was reduced, with the former situation being more frequent than the latter [22].

At M’Baïki, the variations of 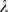 due to the choice of the class width were negligible with respect to the sampling variability of the estimate of 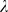 (i.e. the uncertainty on the estimate of 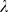 due to the finiteness of the data available). Therefore, at M’Baïki, it can be concluded that the choice of the class width (in the range 1–10 cm) had little importance to estimate the species-specific population growth rates 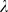. The sampling uncertainty on the estimate of 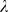 may have been overlooked in matrix modeling (but see [49], [53]–[56]). For instance, Ramula and Lehtilä [9] gave the example of a change of 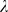 with matrix dimension by 0.006 for a *Primula veris* population and insisted on the difference in population size that this change induces in 50 years, while the standard error on the estimate of 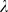 was 0.026, which induces an uncertainty on the population size after 50 years that is much larger. When comparing the 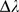 values between tree and herbaceous species, these authors also considered only the between-species variability in 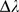 and disregarded all the within-species uncertainty on the estimates of 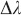. Zuidema [22] also presumably considered only the between-species variability and disregarded the within-species uncertainty on 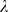 when comparing the values of 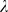 for different class widths (but the exact test was not specified). Enright et al. [8] did not consider the sampling uncertainty on 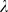, as if transition rates were known exactly.

### Are Matrix Models Reconcilable with other Models?

The way transition matrices are collapsed seems to influence the dependence of the population growth rate 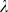 on matrix dimensionality. The greatest dependence of 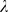 on matrix dimensions was observed when matrices were collapsed using (1) [8], [9]. Equation (1) implicitly means that transition rates are estimated as proportions using data from the class of interest only, i.e. using the maximum likelihood estimator of the underlying Markov chain [57]. However, this proportion estimator of transition rates is known to have a large variance [58]. When compared to other modeling approaches like integral projection models, matrix models may then be expected to under-perform in terms of the precision of predictions [9], [13]. Moreover, equation (1) to collapse transition matrices has the limitation that it depends on the distribution of individuals among classes. This may lead to undesirable results, e.g. the magnitude of changes in 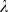 with reduced matrix dimensionality is affected by the distance from the stable class distribution [9]. Theoretically, 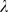 depends on the transition matrix alone and not on the current distribution of individuals among classes [3].

Using (7) to collapse transition matrices implicitly means that transition rates are estimated on the basis of individual-based regressions for growth, mortality and recruitment over the entire size range [7]. In particular, with this approach, the number of parameters to estimate does not depend on the number of classes, which means that the variability of predictions does not depend on matrix dimension (contrary to [13]). As pointed out by Zuidema et al. [7], the use of regressions over the entire size range to estimate transition rates is also the basis of integral projection modeling, thus establishing a close connection between matrix models and integral projection models (IPM). In the same way as the Usher matrix model (5) is a discrete scheme to solve the continuous McKendrick equation (2), the numerical calculation of continuous-size IPM requires some discretization whose expression is a big transition matrix model [7], [12]. In the same way as the discretization of the McKendrick equation into the Usher matrix model brings an error, the discretization of the IPM into a transition matrix model brings an error [7]. In fact, matrix models, IPM and the Fokker-Planck equation (depending on whether size and time are discrete or continuous) are equivalent in some limit [14], [15], with the implication that all estimates that are dependent on class width (like age, see [22]) should tend to the same value when class width is small enough.

Another lesson to learn from this approach is that, although much attention has been devoted to the influence of the class width on predictions (i.e. the discretization of size), the time step (i.e. the discretization of time) may also influence the predictions. At M’Baïki, we collapsed bisannual transition data into annual transition rates so that the matrix model with the smallest class width be close to the McKendrick equation. Although the choice of the time step may bias predictions in the same way as the choice of the class width, and although changing the time step of a matrix model raises issues that are similar to collapsing its dimension [59], the influence of the time step in IPM and matrix models does not seem to have been studied.

### Conclusion

We concur to conclude that matrix models should be used with narrow size classes, to be nearly equivalent with a continuous-size McKendrick equation [7]. The use of regressions over the entire size class and of equations (7) to estimate transition rates allows the modeler to decrease the class width 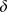, with the only constraint on the lower bound of 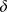 that condition (8) must be met. At M’Baïki like in other studies [8], [9], [22], the choice of the matrix dimensionality had little influence on the population growth rate 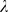. We showed that this influence was similar to that of a diffusion process, and did not act as a systematic bias towards fast pathways. Moreover, the change of 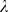 due to the class width was much less than the sampling uncertainty on the estimate of 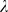. Therefore, the bias of 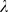 due to matrix dimensionality is not the only statistic to consider; the variance of the estimator of 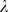 should be considered as well. Making a parallel with the histogram that is more often used than continuous kernel estimators to estimate the density of distribution from a sample of data, searching for a trade-off between bias and sampling variance might lead to matrix models with size classes that are not so narrow.

## Supporting Information

Table S1 Characteristics of population dynamics for 53 tree species at M’Baïki, Central African Republic.
(PDF)
