# Supplementary material for: Matrix Models for Size-Structured Populations: Unrealistic Fast Growth or Simply Diffusion?
Source: PLoS One. 2014 Jun 6;9(6):e98254. doi: 10.1371/journal.pone.0098254 (PMC4048208; doi:10.1371/journal.pone.0098254)
Supplement: Table S1 — Characteristics of population dynamics for 53 tree species at M’Baïki, Central African Republic. (PDF) [file pone.0098254.s001.pdf]

Supporting Information

Matrix models for size-structured populations: unrealistic fast growth or simply diffusion?

Nicolas Picard, Jingjing Liang

Table S1. Characteristics of population dynamics for 53 tree species at M'Baïki, Central African Republic.

The species nomenclature follows the African Plants Database (version 3.4.0) of the Conservatoire & Jardin botaniques de la Ville de Genve, Switzerland and South African National Biodiversity Institute, Pretoria (retrieved December 2013 from `http://www.ville-ge.ch/musinfo/bd/cjb/africa/`); N is the number of observations; a is the dbh growth rate in $\mathrm{cm\,yr^{-1}}$; σ_a is the standard error of the dbh growth rate in $\mathrm{cm\,yr^{-1}}$; r is the recruitment rate in $\%\,\mathrm{yr^{-1}}$; 'model' is the model used for mortality; α, β (in $\mathrm{cm^{-1}}$) and γ (in $\mathrm{cm^{-2}}$ for model (11) and in $\mathrm{cm^{-1}}$ for (12)) are the parameters of the mortality model while σ_α, σ_β and σ_γ are their standard error; AIC is the Akaike Information Criterion of the fitted mortality model; 'variation' is the sign of variation of the population growth rate λ with the class width δ; $\lambda_{\min}$ is the minimum value of λ for $1 \leq \delta \leq 10$ cm; $\lambda_{\max}$ is the maximum value of λ for $1 \leq \delta \leq 10$ cm; λ_{low} is the lower bound of the 95% confidence interval of the estimate of λ for $\delta = 1$ cm while λ_{up} is its upper bound.

Species	Family	N	a	σ_a	r	Model	α	σ_α	β	σ_β	γ	σ_γ
Celtis zenkeri Engl.	Ulmaceae	7295	0.263	0.297	1.028	Eq. (10)	−5.3481	0.2715	0.0261	0.0093		
Staudtia kamerunensis Warb.	Myristicaceae	4328	0.099	0.182	1.317	Eq. (10)	−4.6538	0.3539	0.0047	0.0199		
Coelocaryon preussii Warb.	Myristicaceae	3880	0.155	0.222	1.353	Eq. (10)	−5.0710	0.2985	0.0422	0.0121		
Musanga cecropioides R. Br.	Moraceae	1646	1.155	1.049	7.685	Eq. (11)	−0.3525	0.2876	−0.1045	0.0184	0.0012	0.0002
Carapa procera DC.	Meliaceae	2698	0.081	0.148	1.853	Eq. (10)	−4.4882	0.4953	0.0819	0.0348		
Garcinia punctata Oliv.	Clusiaceae	2774	0.147	0.173	1.802	Eq. (10)	−5.2104	0.4602	0.0984	0.0268		
Dasylepis seretii De Wild.	Flacourtiaceae	2705	0.043	0.111	1.035	Eq. (10)	−5.1364	0.3896	0.0744	0.0255		
Trichilia rubescens Oliv.	Meliaceae	1644	0.098	0.162	2.676	Eq. (10)	−4.6965	0.6206	0.1156	0.0463		

Species	Family	N	a	σ_a	r	Model	α	σ_α	β	σ_β	γ	σ_γ
Rinorea oblongifolia (C.H. Wright) Marquand ex Chipp	Violaceae	1720	0.077	0.116	1.453	Eq. (11)	−19.1948	6.7954	2.1208	0.9793	−0.0724	0.0347
Pycnanthus angolensis (Welw.) Warb.	Myristicaceae	1616	0.237	0.316	1.609	Eq. (10)	−4.0198	0.5445	−0.0207	0.0297		
Pancovia laurentii (De Wild.) Gilg ex De Wild.	Sapindaceae	1622	0.159	0.170	1.510	Eq. (10)	−4.8250	0.6376	0.0362	0.0337		
Trilepisium madagascariense DC.	Moraceae	1529	0.668	0.573	1.602	Eq. (10)	−4.6786	0.4137	0.0232	0.0102		
Diospyros iturensis (Grke) Letouzey & F. White	Ebenaceae	1656	0.068	0.114	1.057	Eq. (10)	−5.4369	0.6553	0.0615	0.0343		
Petersianthus macrocarpus (P. Beauv.) Liben	Lecythidaceae	1595	0.357	0.371	0.940	Eq. (10)	−5.0563	0.5103	0.0131	0.0116		
Eribroma oblongum (Mast.) Pierre ex A. Chev.	Sterculiaceae	1429	0.228	0.311	1.015	Eq. (10)	−4.0965	0.3585	0.0056	0.0151		
Synsepalum stipulatum (Radlk.) Engl.	Sapotaceae	1577	0.109	0.149	0.380	Eq. (10)	−5.4564	0.8023	0.0370	0.0306		
Trichilia prieurieana A. Juss.	Meliaceae	1134	0.282	0.291	1.808	Eq. (10)	−4.9338	0.7228	0.0049	0.0293		
Scottellia coriacea A. Chev. ex Hutch. & Dalziel	Flacourtiaceae	1316	0.109	0.174	0.646	Eq. (10)	−5.3702	0.6707	0.0358	0.0336		
Drypetes chevalieri Beille ex Hutch. & Dalziel	Euphorbiaceae	939	0.103	0.139	2.609	Eq. (10)	−2.8199	0.6994	−0.0182	0.0517		
Angylocalyx pynaertii De Wild.	Fabaceae	1176	0.129	0.204	0.808	Eq. (10)	−4.5435	0.4655	0.0142	0.0165		
Manilkara mabokensis Aubrv.	Sapotaceae	1114	0.226	0.279	1.302	Eq. (10)	−4.0636	0.3716	−0.0040	0.0112		
Manilkara pellegriniana Tisser. & Sillans	Sapotaceae	1020	0.278	0.268	1.373	Eq. (10)	−6.1720	0.7656	0.0403	0.0203		
Polyalthia suaveolens Engl. & Diels	Annonaceae	945	0.211	0.221	1.111	Eq. (10)	−3.7916	0.5480	0.0071	0.0249		
Pausinystalia macroceras (K. Schum.) Pierre	Rubiaceae	808	0.131	0.183	1.795	Eq. (10)	−4.9814	0.5839	0.0748	0.0289		
Entandrophragma cylindricum (Sprague) Sprague	Meliaceae	948	0.369	0.550	0.211	Eq. (10)	−5.2573	0.5280	0.0114	0.0059		
Macaranga paxii Prain	Euphorbiaceae	417	0.718	0.700	6.235	Eq. (10)	−2.8153	0.3873	0.0231	0.0141		
Celtis mildbraedii Engl.	Ulmaceae	908	0.461	0.426	0.165	Eq. (10)	−5.9599	0.6876	0.0448	0.0119		
Guarea laurentii De Wild.	Meliaceae	712	0.120	0.227	1.756	Eq. (10)	−3.7132	0.4535	0.0128	0.0223		
Diospyros canaliculata De Wild.	Ebenaceae	659	0.095	0.143	2.124	Eq. (10)	−4.1195	0.9494	0.0597	0.0642		
Drypetes gilgiana (Pax) Pax & K. Hoffm.	Euphorbiaceae	569	0.097	0.148	3.515	Eq. (10)	−2.5370	0.9925	−0.0582	0.0792		
Cola lateritia K. Schum.	Sterculiaceae	760	0.151	0.228	1.316	Eq. (10)	−5.7544	0.6513	0.0432	0.0142		
Strombosia grandifolia Hook. f.	Olacaceae	855	0.250	0.263	0.643	Eq. (11)	−17.0185	12.4065	0.9162	0.9922	−0.0174	0.0194
Dialium guineense Willd.	Caesalpiniaceae	675	0.100	0.245	1.481	Eq. (11)	−5.6322	1.3007	0.2035	0.1259	−0.0033	0.0024
Diospyros crassiflora Hiern	Ebenaceae	724	0.168	0.215	0.829	Eq. (10)	−3.8688	0.5047	−0.0021	0.0158		
Corynanthe pachyceras K. Schum.	Rubiaceae	744	0.118	0.174	0.739	Eq. (10)	−4.7796	1.0131	0.0062	0.0473		
Triplochiton scleroxylon K. Schum.	Sterculiaceae	560	0.431	0.548	1.429	Eq. (12)	−3.1450	0.3643	−0.3923	0.3486	−0.0720	0.0374

Species	Family	N	a	σ_a	r	Model	α	σ_α	β	σ_β	γ	σ_γ
Lecaniodiscus cupanioides Planch.	Sapindaceae	742	0.168	0.218	0.876	Eq. (10)	−4.7941	0.9735	0.0073	0.0463		
Anonidium mannii (Oliv.) Engl. & Diels	Annonaceae	748	0.220	0.267	0.735	Eq. (10)	−4.6141	0.8247	−0.0084	0.0306		
Cola nitida (Vent.) Schott & Endl.	Sterculiaceae	703	0.084	0.153	0.498	Eq. (10)	−3.8671	0.9890	−0.0201	0.0518		
Funtumia elastica (P. Preuss) Stapf	Apocynaceae	507	0.267	0.284	2.170	Eq. (10)	−4.3292	0.8591	0.0200	0.0298		
Santiria trimera (Oliv.) Aubrv.	Burseraceae	684	0.202	0.283	1.096	Eq. (10)	−1.3022	1.6091	−0.2037	0.1186		
Pouteria altissima (A. Chev.) Baehni	Sapotaceae	537	0.306	0.389	1.210	Eq. (10)	−3.5400	0.5624	0.0027	0.0239		
Chrysophyllum africanum A. DC.	Sapotaceae	678	0.338	0.409	1.180	Eq. (10)	−5.3572	0.6464	0.0253	0.0133		
Celtis adolfi-friderici Engl.	Ulmaceae	701	0.278	0.302	0.499	Eq. (10)	−4.8007	0.7364	0.0348	0.0266		
Strombosiopsis tetrandra Engl.	Olacaceae	697	0.193	0.201	0.861	Eq. (10)	−6.0990	1.2688	0.0699	0.0573		
Aubrevillea kerstingii (Harms) Pellegr.	Mimosaceae	610	0.153	0.285	1.311	Eq. (10)	−0.0594	5.0376	−0.4546	0.4412		
Drypetes sp.	Euphorbiaceae	537	0.127	0.176	1.955	Eq. (10)	−3.2990	0.9981	−0.0569	0.0639		
Ricinodendron heudelotii (Baill.) Pierre ex Heckel	Euphorbiaceae	418	0.681	0.743	3.349	Eq. (12)	−1.1118	0.7647	−0.2291	0.0739	−0.0303	0.0058
Entandrophragma angolense (Welw.) C. DC.	Meliaceae	578	0.263	0.323	1.211	Eq. (11)	−3.1176	1.6839	−0.1195	0.1098	0.0010	0.0007
Drypetes obanensis S. Moore	Euphorbiaceae	567	0.137	0.265	1.146	Eq. (10)	−4.2896	0.5612	0.0231	0.0225		
Khaya anthotheca (Welw.) C. DC.	Meliaceae	537	0.286	0.444	0.745	Eq. (12)	−1.2746	0.8177	−0.8515	0.2863	−0.0624	0.0151
Albizia glaberrima (Schumach. & Thonn.) Benth.	Mimosaceae	371	0.578	0.616	2.561	Eq. (10)	−2.5271	0.4247	−0.0160	0.0099		
Chrysophyllum lacourtianum De Wild.	Sapotaceae	437	0.389	0.390	1.602	Eq. (10)	−2.7091	1.4331	−0.1079	0.0865		

Species	AIC	variation	λ_{min}	λ_{max}	λ_{low}	λ_{up}
Celtis zenkeri	730.8	increasing	1.004513	1.004565	1.002121	1.007088
Staudtia kamerunensis	495.3	decreasing	1.007077	1.008400	1.005756	1.011139
Coelocaryon preussii	579.9	increasing	1.006039	1.006130	1.002482	1.009446
Musanga cecropioides	1145.2	decreasing	1.005201	1.011443	0.998973	1.022691
Carapa procera	781.1	increasing	1.001685	1.002529	0.996283	1.007569
Garcinia punctata	637.7	increasing	1.003784	1.004806	0.998140	1.009784
Dasylepis seretii	431.6	decreasing	1.001201	1.002502	0.998832	1.010323
Trichilia rubescens	533.0	increasing	1.006509	1.007723	0.998550	1.015163
Rinorea oblongifolia	260.9	increasing	1.008902	1.012476	1.005211	1.013015
Pycnanthus angolensis	218.9	increasing	1.011100	1.011551	1.005606	1.014948
Pancovia laurentii	252.8	increasing	1.006900	1.006968	1.000958	1.013352
Trilepisium madagascariense	294.5	increasing	1.002888	1.003161	0.994334	1.009534
Diospyros iturensis	217.5	increasing	1.004725	1.004818	0.999745	1.009521
Petersianthus macrocarpus	181.9	increasing	1.003647	1.003686	0.999202	1.008252

Species	AIC	variation	λ_{min}	λ_{max}	λ_{low}	λ_{up}
Eribroma oblongum	263.7	decreasing	1.001389	1.001591	0.994063	1.006296
Synsepalum stipulatum	181.3	increasing	0.997354	0.997783	0.992652	1.002056
Trichilia prieurieana	109.0	decreasing	1.014023	1.014026	1.008219	1.020554
Scottellia coriacea	130.2	increasing	1.000855	1.001019	0.995679	1.006031
Drypetes chevalieri	347.0	increasing	1.004006	1.004106	0.995827	1.014129
Angylocalyx pynaertii	181.1	increasing	1.000636	1.000693	0.995912	1.005736
Manilkara mabokensis	179.8	nearly constant	1.005429	1.005430	1.000068	1.011591
Manilkara pellegriniana	74.6	decreasing	1.009855	1.009939	1.003895	1.016759
Polyalthia suaveolens	227.6	increasing	0.997757	0.997809	0.989335	1.005414
Pausinystalia macroceras	178.6	increasing	1.006221	1.006639	0.997469	1.015389
Entandrophragma cylindricum	102.5	increasing	0.994516	0.994890	0.987945	1.000472
Macaranga paxii	260.5	increasing	1.018550	1.018911	0.995802	1.040807
Celtis mildbraedii	167.1	increasing	0.978537	0.982309	0.804119	0.988585
Guarea laurentii	193.1	increasing	1.002931	1.002971	0.994624	1.012032
Diospyros canaliculata	209.3	increasing	1.003139	1.003666	0.992558	1.015258
Drypetes gilgiana	183.2	decreasing	1.016856	1.016953	1.005180	1.030374
Cola lateritia	94.6	decreasing	1.009010	1.009066	1.002090	1.017024
Strombosia grandifolia	32.0	increasing	1.005698	1.005820	1.002447	1.010012
Dialium guineense	183.0	increasing	1.000004	1.000897	0.999900	1.010478
Diospyros crassiflora	142.2	increasing	0.998688	0.998690	0.992046	1.005246
Corynanthe pachyceras	83.2	increasing	1.002513	1.002517	0.996434	1.008705
Triplochiton scleroxylon	131.4	increasing	1.007108	1.007184	0.995547	1.017731
Lecaniodiscus cupanioides	83.2	increasing	1.003700	1.003704	0.996515	1.010326
Anonidium mannii	73.8	decreasing	1.003782	1.003801	0.997419	1.008800
Cola nitida	108.8	increasing	0.999854	0.999926	0.992569	1.003247
Funtumia elastica	109.6	increasing	1.011629	1.011638	1.001916	1.023646
Santiria trimera	94.2	decreasing	1.006164	1.007523	1.003683	1.012648
Pouteria altissima	147.9	increasing	0.996687	0.996705	0.984690	1.007967
Chrysophyllum africanum	79.0	increasing	1.005070	1.005261	0.996485	1.013453
Celtis adolfi-friderici	131.9	increasing	0.989250	0.990719	0.977422	1.002754
Strombosiopsis tetrandra	71.6	increasing	1.000674	1.001235	0.990261	1.009799
Aubrevillea kerstingii	28.8	increasing	1.012144	1.013115	1.006699	1.018644
Drypetes sp.	86.2	decreasing	1.011961	1.012205	1.004405	1.021823
Ricinodendron heudelotii	151.7	increasing	1.017408	1.017427	0.999377	1.034851
Entandrophragma angolense	57.1	decreasing	1.008701	1.009360	1.003400	1.016407
Drypetes obanensis	119.4	increasing	1.000622	1.000777	0.990428	1.010086

Species	AIC	variation	λ_{min}	λ_{max}	λ_{low}	λ_{up}
Khaya anthotheca	54.2	increasing	0.999993	1.000408	0.986382	1.009305
Albizia glaberrima	126.4	increasing	1.003777	1.003886	0.995371	1.017408
Chrysophyllum lacourtianum	47.1	decreasing	1.012218	1.013011	1.006011	1.023470

Models for mortality:

$$m(x)\tau = \text{logit}^{-1}(\alpha + \beta x) \tag{10}$$
$$m(x)\tau = \text{logit}^{-1}(\alpha + \beta x + \gamma x^2) \tag{11}$$
$$m(x)\tau = \text{logit}^{-1}(\alpha + \beta x \exp(\gamma x)) \tag{12}$$
